# Exploring the Relationship between Callous-Unemotional Traits and Implicit Attitudes toward Violence

**DOI:** 10.3390/healthcare11101445

**Published:** 2023-05-16

**Authors:** Antonio Olivera-La Rosa, Omar Amador, Erick G. Chuquichambi, César Andrés Carmona-Cardona, Sergio Andrés Acosta-Tobón, Olber Eduardo Arango-Tobón, Javier Villacampa

**Affiliations:** 1 Department of Psychology and Social Sciences, Universidad Católica Luis Amigó, Transversal 514A #67B 90, Medellín 050034, Colombia; 2 Human Evolution and Cognition Group, University of the Balearic Islands—CSIC, Carretera de Valldemossa, km 7.5, 07122 Palma de Mallorca, Spain; 3 Institute Psicoeducativo from Colombia, Medellín 050034, Colombia

**Keywords:** psychopathy, callous-unemotional traits, young offenders, violence

## Abstract

Past research has associated *callous-unemotional traits* (CU) in young people with serious conduct problems and antisocial behavior. However, whether CU traits influence implicit attitudes toward violence remains largely unexplored. We assess this hypothesis in two independent samples: a sample of youth with no criminal records (Study 1, N = 86), and in a sample of young offenders (Study 2, N = 61). Both groups were not compared due to theoretical (very different demographics) and statistical reasons (the total sample was insufficient to be able to reach the statistical power required in the comparison of both groups). Further, we use an implicit procedure to examine whether CU traits modulate wanting for violent stimuli. Across two samples of youth, we found little evidence of an association between CU traits and implicit violent cognition. In youth with no criminal records, implicit attitudes toward violence were related to the unemotional factor of CU traits, but unrelated to other factors and to a global CU traits score. CU traits were not associated with implicit attitudes toward violence in young offenders. The latter finding was mirrored in the implicit wanting task. Overall, our findings cast some doubts on the adequacy of implicit measures to assess implicit violent cognition in youth with CU traits. We discuss potential methodological limitations of this research (e.g., characteristics of the sample and performance in the implicit procedures) that may impact our results.

## 1. Introduction

CU traits (e.g., lack of empathy, lack of guilt, failure to put effort in important activities, and poverty in emotional expressions) are associated with a severe and persistent trajectory of antisocial, delinquent, violent, and aggressive behavior [1,2,3]. Although CU traits have been mostly associated with premeditated and instrumental violent behavior [4], research in community samples suggests that CU traits might be associated with a combination of instrumental and non-instrumental types of aggression [5]. Some authors conceptualized CU traits as the affective component of the construct of psychopathy in adults [6], being a “downward” extension of adult psychopathy to youth [7]. Evidence from a meta-analysis indicated that youth with higher CU traits exhibited lower empathy [8]; that is, individuals with CU traits are less likely to be inhibited from acting aggressively by the aversive experience associated with hurting other individuals [9]. For instance, CU traits predicted both the frequency of gun carrying at the first arrest and the likelihood of using a gun during a crime in the 48 months after the first arrest [10]. Consistent with these findings, the Diagnostic and Statistical Manual of Mental Disorders Fifth Edition (DSM-5) included the specifier “with limited prosocial emotions”, within the diagnosis of conduct disorder to designate those youths with elevated CU traits [11].

Although the link between CU traits and violent behavior is consistent in the literature, implicit attitudes toward violence remain understudied in youths with CU traits. This is somewhat surprising since attitudes toward violence play a crucial role in the translation of hostile feelings into aggressive behaviors [12,13,14]. Indeed, the limited introspection capacity of the participants [15] and the social desirability bias may compromise the use of self-report instruments [16,17], which supports the need to incorporate implicit measures that complement established self-reports in “sensitive” contexts, such as violent cognition [12,18,19,20]. For instance, the DSM-5 indicates that individuals with conduct disorder with the “limited prosocial emotions” specifier might not readily admit to the traits in a self-report [11]. 

A measure is regarded as implicit when it is produced by the psychological attribute to be measured (e.g., attitude) in conditions typically associated with automaticity [21]. Implicit methods are distinguished by being “indirect”; that is, the mental content is inferred from behavioral responses (e.g., reaction times, RT). Therefore, responses are assumed to be more difficult to manipulate than self-reports [22]. When it comes to assessing spontaneous behaviors and socially sensitive constructs, implicit measures can show superior predictive validity than explicit measures [16,23].

The literature on implicit attitudes toward violence is still scarce. Robertson and Murachver [24] found that an incarcerated sample evidenced more tolerant implicit attitudes toward violence than the non-incarcerated sample, but that this difference was less evident when measured explicitly (for a similar finding see [25]). Compared to a control group, men registered in a treatment for intimate partner violence exhibited more positive implicit attitudes toward violence [26]. More recently, Olivera-La Rosa and colleagues [27] found that young offenders with conduct disorder showed less negative (more positive) implicit attitudes toward physical violence than the group of offenders without conduct disorder. 

Research on the relationship between implicit attitudes toward violence and psychopathy has found mixed results. Snowden and colleagues [28] found that, relative to non-murderers, murderers highest in psychopathy evidenced a reduced negative implicit association to violence. However, they did not find any influence of psychopathy in the non-murdering group. Zweets and colleagues [17] showed that implicit attitudes toward violence were related to the antisocial facet of psychopathy (e.g., juvenile delinquency), but unrelated to other psychopathy facets (e.g., callous and lack of empathy), and also unrelated to a global psychopathy score. In this vein, Suter and colleagues [29] found a limited association between psychopathic traits and implicit attitudes toward transgression and aggression in an offender group. Overall, these findings suggest that the relationship between psychopathy and implicit attitudes toward violence may be rather complex and require further research.

So far, implicit research on attitudes toward violence has largely focused on associations between violence and valence (good vs. bad). However, previous studies showed that people can have multiple representations of preference toward an object [30], even at an implicit level [31]. Following the proposal of Blumenthal and colleagues [12], implicit measures of violent cognitions should distinguish between evaluations of valence (e.g., whether violence is good or bad) to identify “liking” (e.g., whether one enjoys/likes violence) or “wanting” (e.g., whether one is motivated to obtain and consume a stimulus) [32] responses. For instance, even though violence is considered a “bad” thing even among offender populations [28], these same people might also want violent stimuli (“I know violence is bad, but I still want it”). Further, although people typically want what they like and like what they want, some research suggests that liking and wanting are different phenomena that can be dissociated in some cases [30]. More importantly, implicit wanting may be a better predictor of behavior than self-reports in areas likely to be influenced by social desirability bias [32]. 

The present study aims to examine whether CU traits influence implicit attitudes toward violence in two samples of youths with different characteristics: youth with no criminal records (Study 1) and young offenders (Study 2). Further, we assess whether CU traits are related to wanting for violent stimuli. Since we have no evidence, so far, of the existence of specific effects of CU traits in implicit violent cognition, we hypothesized that:

**H1:**  *In both groups, CU traits will be associated with less negative implicit attitudes toward violence.*

**H2:**  *In both groups, CU traits will influence participants’ implicit wanting for violent stimuli.*

## 2. Study 1: Sample of University Students

### 2.1. Method

#### 2.1.1. Participants: Sample of University Students

We conducted this study in a sample of university students from a Colombian university. We chose a sample consisting of these characteristics because we considered it important to study the role of CU traits in violent cognition in a sample that is not involved in criminal behavior. Thus, we recruited one hundred and two students from a Colombian university (41 males, age: *M* = 20.98, *SD* = 5.13). All participants identified themselves as Latino. The included participants were undergraduate students that pertain to different socioeconomic levels (strata 1: 8.29%, strata 2: 33.45%, strata 3: 43.96%, strata 4:11.31%, strata 5: 2.86%, strata 6: 0.12%) (Table S1: Demographics). We ran an a priori power analysis using the pwr package in R [33] to determine the adequate sample for our study. We defined an alpha level of 0.05 and a small effect on participants’ attitudes toward violence (valence D scores) based on previous studies [27]. Results indicated that 40 participants would have a statistical power of 80% to reach the expected effect size.

Randomized sampling was not applied as we included every individual fulfilling the main inclusion criterion (university students who had normal or corrected-to-normal vision). The clinical history and criminal records of all participants were considered to exclude any subject with evidence of any medical condition that may suggest the existence of any other developmental, emotional, or behavioral disorder. None of the participants who accepted to be a part of the study was excluded. A non-probability sampling was used for this study. All participants were invited via mail and social networking to join the study as a part of their course credits; therefore, the participants did not get paid for the participation. The study was conducted in the psychology laboratory of the university during August of 2022. The procedure was always supervised by two of the authors of this research. All participants had normal or corrected-to-normal vision. Ethics approval was provided by Luis Amigo Catholic University Institutional IRB (No. 65611/2022). Informed consent was obtained from each participant.

#### 2.1.2. Materials and Procedure

Participants completed the Inventory of Callous-Unemotional Traits (ICU) [34,35], which has been widely applied to assess CU traits in children, adolescents, and adults [36] (Table S2: ICU items). Participants completed the ICU in the psychology laboratory of the university under the supervision of two of the authors of this research. The ICU considers three reporting versions: self-report, teachers, and parents. Although the ICU was initially developed as a single scale, it is now widely viewed as including an unemotional factor (e.g., “I hide my feelings from others”), an uncaring factor (e.g., “I care about how well I do at school or work”) and a callous factor (e.g., “I do not care who I hurt to get what I want”) [37]. Essau et al. [38] already showed that a three-factor bifactor model (callousness, uncaring, and unemotional) provided acceptable fit to the data of a large sample of adolescents. Subsequently, Kimonis and colleagues [4] found that a three-factor bifactor structural model of the general and specific factors of the Inventory of Callous–Unemotional Traits also had an adequate fit to the data in a sample of juvenile offenders. More recent studies also showed a similar three-factor structure to prior studies conducted with youth [35,36]. Recent meta-analyses have provided support for its ability to measure CU traits continuously [3], although some studies indicate that the unemotional factor is weakly correlated with the CU construct and callousness and uncaring factors [36,37].

After completing the ICU, participants were asked to complete the Single Target Implicit Association Test (ST-IAT) [39] to assess implicit attitudes toward violence. We displayed the stimuli on a 20-inch screen (60 Hz screen refresh rate) with a PC running OpenSesame v.3.0.7 [40] on Windows 8 (Microsoft Corporation, Redmond, WA, USA). The procedure took place in the psychology laboratory of the university and was always supervised by two of the authors of this research. In the ST-IAT, participants were asked to categorize each presented stimulus as quickly and accurately as possible. We assessed the association of the target category (e.g., violence) toward positive and negative valence attribute categories: shorter response latencies are indicative of easier stimuli/category assignment (i.e., lesser interference/more compatibility), which is indicative of stronger implicit associations (for a detailed description of the procedure see [27]). 

Following the same procedure as Bluemke and Friese [41] for category assignment and stimulus proportions across ST-IAT blocks, each stimulus was presented at least twice, adding up to 72 trials (Table S3). The concept of violence (target category) was represented by 4 pictures from the International Affective Picture System (IAPS) that [42] selected for their violent content. In the valence ST-IAT (i.e., associations between violence and valence), the concepts of “good” and “bad” were represented by four “good” words (e.g., feliz [happy]) and four “bad” words (e.g., veneno [poison]). All stimuli were selected from Blumenthal and colleagues [12] (Table S4). The categorization task opened with sixteen trials for the training block, preceding the first combined block. This practice block only considered two categories (“good/bad”) and the obtained scores were not considered in further analysis. The sequence of the item/category assignment was randomized. 

Next, participants completed the implicit wanting task (adapted from [43]). In this task, participants hit a computer key in response to a picture. Presentation time was decided by participants who had to voluntarily press a key for as long as they wished to see the target image (e.g., a violence picture) on screen. The rationale behind this task is that participants would make a bigger physical effort (continuous key pressing) if they wanted to visually consume the violence stimuli. We used the same 4 pictures as in the ST-AIT as violence targets (the name of these items is available in Table S4). Every trial started with a fixation figure that appeared on the screen until the participant pressed the spacebar. Next, the violence target was presented, and it remained on screen for as long as the participant continuously pressed the spacebar. When the spacebar was released, the violence target was replaced by the fixation figure, signaling the start of a new trial. Participants received the following instructions: ‘When the fixation figure appears, you have to press the spacebar and you’ll see an image. You can see it as long as you want, while you continue pressing the spacebar’ (for a detailed description of the procedure see [43]). 

### 2.2. Results

#### Data Analysis

We carried out analyses within the R environment for statistical computing [33]. Following previous ST-IAT research [41], participants with error rates above 30% were dropped from the analysis (n = 16). Delayed responses (RTs > 3000 ms) were also removed from the analysis (230 trials). As a result, our final analysis was based on a sample size of 86 participants (35 male). Two linear regression analyses were conducted to model whether the participants’ ICU scores modulated valence D scores and wanting RTs. ICU scores showed acceptable internal consistency reliability (Cronbach’s α = 0.76, M = 23.64, SD = 6.95). This is consistent with previous research showing that the ICU showed an acceptable internal consistency for its total score (Cronbach’s α= 0.77), and the callousness (α= 0.70) and uncaring (α= 0.73) subscales. The unemotional dimension showed the worst estimate (α= 0.64) and the lowest correlations with the total score and the other subscales (callousness Pearson’s ρ = 0.25; uncaring ρ = 0.09) [4]. 

Similarly, we conducted two multiple regression models to examine whether the scores in the ICU dimensions (callousness, uncaring, unemotional) also modulated valence D scores, and wanting responses. Correlations between the ICU dimensions were low (callousness and uncaring: *r_p_* (Pearson) = 0.29; callousness and unemotional: *r_p_* = 0.26; uncaring and unemotional: *r_p_* = 0.32). A final regression model examined the relationship between valence D scores and wanting responses.

We interpreted D scores following the standard magnitude of strength adopted in previous research [44]. Participants evidenced small-to-moderate negative attitudes toward violence (D = 0.29). Linear regression results are presented in Table 1. ICU scores did not significantly influence either valence scores or wanting responses (Mean viewing time = 770 ms, SD = 365 ms). Participants who scored higher in the unemotional facet showed higher valence scores. Higher scores in the unemotional factor are indicative of increased negative attitudes toward violence. In contrast, the callousness and uncaring scores did not significantly modulate the participants’ valence D. Similarly, none of the ICU factors had a significant effect on wanting responses. The relationship between valence and wanting responses was also non-significant. 

## 3. Study 2: Sample of Young Offenders

### 3.1. Method

#### 3.1.1. Participants: Sample of Young Offenders

We conducted this study in a sample of young offenders in a large Colombian city. We considered that testing the relationship between CU traits and implicit violent cognition in a sample consisting of these characteristics will extend the scope of our results and, therefore, would provide a deeper characterization of the role of CU traits in implicit violent cognition. However, we were aware that testing our hypothesis in such different samples (Study 1: university students and Study 2: young offenders) requires treating both samples as independent samples (see Limitations section). The minimum sample size was defined following the power analysis conducted in Study 1. We recruited 70 male young offenders from a total sample of 120 young offenders (age: M = 16.84, SD = 0.86). A total of 50 individuals were not considered in the study due to psychiatric conditions, abstinence syndrome, or the lack of desire to participate. All participants were Colombian and identified themselves as Latino. The fact that only male participants were part of the study is because, at the moment when data were collected, only male offenders were in the young offenders’ center. Although participants were not asked to report their socioeconomic level, the data available from the offender center indicate that participants who were part of the study are in a low socioeconomic level (strata 1–2 Colombia, which are the two lowest socioeconomic levels) and do not have a higher education (Table S1: Demographics). 

Randomized sampling was not applied as we included every individual fulfilling the main inclusion criterion (young offenders who had normal or corrected-to-normal vision). The clinical history and criminal records of all participants were studied to exclude any subject with evidence of any medical condition that may suggest the existence of any other developmental, emotional, or behavioral disorder. The recruitment process for these participants was as follows: First, we contacted the young offenders’ center to inform them of the objective and scope of the study. Once the authorization was obtained from the director of the institution and from the legal representatives of the young offenders, we proceeded to invite all young offenders who were present during the period data were collected. We notified all participants that participation was voluntary and would have no influence on their legal involvement. Experimental sessions took place in a young offenders’ center in a large Colombian city during May and June of 2022. The procedure was always supervised by one of the authors of this research. The study protocol was approved by Luis Amigo Catholic University Institutional IRB (No. 65611/2022). Informed consent was obtained from each participant and their legal guardian (the principal of the institution). 

#### 3.1.2. Materials and Procedure

All relevant methodological variables were held constant with Study 1. First, participants completed the ICU [34,35]. Next, they were asked to complete the ST-IAT [39] and the implicit wanting task [43] to assess implicit violent cognition. 

### 3.2. Results

#### Data Analysis

We carried out analyses within the R environment for statistical computing (Champely, 2020). As in Study 1, participants with error rates above 30% were dropped from the analysis (n = 9). Delayed responses (RTs > 3000 ms) were also removed from the analysis (157 trials). Therefore, our final analysis was based on a sample size of 61 participants (61 male). As in Study 1, linear regressions were conducted to model whether ICU scores and ICU dimensions (i.e., callousness, uncaring, unemotional) modulated valence D scores, wanting responses, and the relationship between these variables. As in Study 1, ICU scores showed acceptable internal consistency reliability (Cronbach’s α = 0.70, M = 24.20, SD = 7.06). Similarly, correlations between the ICU dimensions were low (callousness and uncaring: *r_p_* = 0.12; callousness and unemotional: *r_p_* = −0.011; uncaring and unemotional: *r_p_* = 0.013).

As expected, participants evidenced only small negative attitudes toward violence (D = 0.11). Linear regression results are presented in Table 2. As in Study 1, ICU scores did not significantly modulate either valence scores or wanting responses (Mean viewing time = 1000 ms, SD = 555 ms). However, contrary to Study 1, the unemotional factor did not significantly modulate the participants’ valence D. Callousness and uncaring also had no significant effects on the participants’ valence D. On the other hand, these ICU subscales had no effect on wanting responses. The relationship between D scores and wanting responses was also non-significant. 

## 4. General Discussion

To the best of our knowledge, the present research constitutes the first attempt to study the relationship between implicit violence cognition and CU traits. It has been suggested that the association between psychopathy and offending relies on traits such as callousness, lack of empathy, and lack of guilt or remorse [45], traits that are at the core of the CU construct [4]. Accordingly, CU traits are strongly associated with violent and aggressive behavior [3]. Postulating that attitudes are important determinants of violent behavior [13,14], it seems theoretically plausible to assume that CU traits will impact attitudes toward violence. Built on these findings, we hypothesized that CU traits would influence attitudes toward violence (H1) and implicit wanting for violent stimuli (H2). We tested these hypotheses in two independent samples: a sample of youth with no criminal records (Study 1) and a sample of young offenders (Study 2). Contrary to our hypotheses, we did not find evidence of a consistent effect of CU traits in violence cognition in two samples of youths with different characteristics. This pattern of results was replicated in the wanting task. Our results add to the list of controversial studies on the relationship between psychopathic traits and implicit attitudes toward violence. Previous studies showed that this relationship is moderated by certain factors, such as whether the participant committed murder or not [20]. The impact of psychopathic traits on implicit attitudes toward violence may be limited or rather complex [30]. For example, Zweets and colleagues [17] found that implicit attitudes toward violence were unrelated to the affective facet of psychopathy, which is consistent with our findings. 

Indeed, our results showed that CU traits do not influence implicit violent cognition. Some authors claim that some offenders have implicit theories of the world (which are not available to conscious access) underlying their violent behaviors [46]. At first glance, our results question this claim, at least when it comes to offenders with CU traits. Implicit measures are assumed to look at early automatic evaluations of attitude objects (e.g., violence). In the context of psychopathy, it has been suggested that some aspects of behavior may be better explained because of controlled processes (more likely to be assessed by explicit measures) while other aspects of behavior may be better characterized as the result of automatic responses (more likely to be assessed by implicit measures) [47]. Therefore, our results support the claim that CU traits are mostly associated with premeditated violent behavior [4], suggesting that CU traits may not be a strong predictor of spontaneous aggressive reactions (which are more likely to be assessed/predicted by implicit measures).

This lack of consistent evidence may be explained by implicit procedures (or at least, IAT-like procedures such as the ST-IAT) having difficulty in measuring the motivational aspect of the affective component of psychopathy. For instance, “knowing” that an object is negative does not imply that the object is not enjoyable or desirable [12]. Previous research with the IAT found that both smokers and nonsmokers can hold negative implicit attitudes to smoking, suggesting that implicit attitudes are not a determinant of smoking behavior [48]. Furthermore, as mentioned previously, research with the IAT showed that even offender populations have a general view that violence is bad [28]. Our results support this finding: across two samples, we found that violence was regarded as a negative thing, even among young offenders. In this vein, some evidence suggests that psychopaths know right from wrong, but do not care about such knowledge or the implications derived from their antisocial behavior [49]. Hence, it can be argued that the valence ST-IAT may be more of a measure of “cold” (cognitive) evaluations of violence (“whether violence is understood as a good/bad thing”), but it failed to capture the appetitive component of global implicit attitudes in participants with elevated CU traits. Indeed, there has been controversy on whether and how implicit measures predict behavior [50], in part because implicit procedures may only measure certain aspects of attitudes [51]. 

Besides ST-IAT, we also include a measure of implicit wanting in our research (H2). Although this hypothesis was exploratory, the finding that CU traits do not predict implicit wanting for violence stimuli nevertheless seemed unexpected, especially in the group of young offenders. Therefore, our data do not support that implicit wanting predicts behavior in sensitive domains [32]), such as violent behavior. An alternative explanation is that participants with elevated CU traits fail to put the effort into the wanting task. As mentioned previously, CU traits are characterized by a failure to put effort in important activities [2]. This particularity is reflected in the uncaring and callousness dimensions of the ICU, which include items like “I do not care about doing things well” or “I always try my best” [38]. It is also possible that our wanting procedure failed to capture the quality of a truly motivational wanting response, which may require that participants experience a state of deprivation [32]. Future research should explore different implicit procedures (e.g., wanting implicit association test, Koranyi et al. [32]) to identify which tasks are more suitable to assess implicit violent cognition in youth with CU traits.

The finding that the unemotional factor of CU traits predicted more negative attitudes toward violence in youth with no criminal records was intriguing. It may be argued that the unemotional factor proved to be poorly correlated with the CU construct [38], which may compromise the interpretation of this particular result. Indeed, the fact that this finding was not replicated in the sample of young offenders suggests that the role of the unemotional factor in implicit attitudes toward violence may depend on certain moderators involved in criminal behavior. However, this possibility is highly speculative and still requires direct empirical investigation.

Several limitations should be noted. First, our sample of young offenders was based exclusively on male offenders, which compromised the possibility of comparing both samples. Although research on young offenders tends to focus on male adolescents [18], future research should address this limitation by assessing implicit attitudes toward violence in a larger sample of female young offenders. Further, the total sample was insufficient to be able to reach the statistical power required in the comparison of both groups (young offenders vs. non-young offenders). In this vein, the fact that both groups present very different characteristics (e.g., sex, age, education) compromised the viability of the comparison. Therefore, we decided to treat both samples as independent samples (Study 1 and Study 2). Second, both samples were based exclusively on Colombian participants, which may compromise the generality of our results in other contexts. In this vein, it may be argued that some ICU subscales’ reliability partially varies according to culture [37]. However, we used a translated version of ICU that was previously applied to Colombian samples [52]. Future cross-cultural research on the association of CU traits and implicit violent cognition should be conducted. Third, we did not control for the type of crime of young offenders, which may have implications for the impact of psychopathic traits on implicit attitudes toward violence [28]. Fourth, we did not include a measure of implicit liking, which evidenced positive associations with official records of convictions in an offender sample [12]. The non-inclusion of this measure was to minimize the fatigue inherent in the categorization task. Despite these limitations, this research extends previous studies on psychopathic traits by revealing that CU traits did not influence implicit attitudes toward violence, as measured by an ST-IAT. We believe that non-significant results are crucial to avoid wasting resources in scientific research, especially in topics as sensitive as the study of psychopathic traits and their implications in violent behavior.

## Figures and Tables

**Table 1:** Sample of university students.

Fixed Effects	*β*	*SE*	*T*	*p*	95% CI
Valence D					
ICU Score	0.0065	0.0065	1	0.32	−0.0065, 0.019
Callousness	0.0060	0.015	0.39	0.70	−0.024, 0.036
Uncaring	−0.021	0.016	−1.31	0.19	−0.054, 0.011
Unemotional	0.030	0.014	2.16	0.034 *	0.0023, 0.058
Wanting (viewing time)					
ICU Score	7.57	5.76	1.31	0.19	−3.88, 19.03
Callousness	7.04	13.72	0.51	0.61	−20.25, 34.32
Uncaring	−2.25	14.60	−0.15	0.88	−31.29, 26.79
Unemotional	16.11	12.48	1.29	0.20	−8.71, 40.93
Valence D~Wanting	0.000082	0.00012	0.66	0.51	−0.00016, 0.00033

Note. * *p* < 0.05.

**Table 2:** Sample of young offenders.

Fixed Effects	*β*	*SE*	*t*	*p*	95% CI
Valence D					
ICU	0.0025	0.0087	0.28	0.78	−0.015, 0.020
Callousness	0.00077	0.014	0.054	0.96	−0.028, 0.029
Uncaring	0.00084	0.015	0.057	0.95	−0.029, 0.030
Unemotional	0.011	0.022	0.50	0.62	−0.033, 0.054
Wanting (viewing time)					
ICU	−6.01	10.71	−0.56	0.58	−27.45, 15.42
Callousness	−9.21	16.76	−0.55	0.58	−42.80, 24.39
Uncaring	−15.25	19.26	−0.79	0.43	−53.85, 23.35
Unemotional	18.37	25.65	0.72	0.48	−33.03, 69.77
Valence D~Wanting	0.000033	0.000067	0.48	0.63	−0.00010, 0.00017

## Data Availability

The data presented in this study are available on request from the corresponding author.

## Supplementary Materials

The following supporting information can be downloaded at: https://www.mdpi.com/article/10.3390/healthcare11101445/s1, Table S1: Demographics; Table S2: Individual items of the ICU; Table S3: Category assignment and stimulus proportions across ST-IAT blocks for an exem-plary participant; Table S4: Attribute and target items.
